# Tonse Pamodzi: Developing a combination strategy to support adherence to antiretroviral therapy and HIV pre-exposure prophylaxis during pregnancy and breastfeeding

**DOI:** 10.1371/journal.pone.0253280

**Published:** 2021-06-25

**Authors:** Lauren M. Hill, Friday Saidi, Kellie Freeborn, K. Rivet Amico, Nora E. Rosenberg, Suzanne Maman, Twambilile Phanga, Mercy Tsidya, Sara Chirwa, Chifundo Zimba, Wilbroad Mutale, Benjamin H. Chi

**Affiliations:** 1 Department of Health Behavior, Gillings School of Global Public Health, University of North Carolina, Chapel Hill, NC, United States of America; 2 UNC Project–Malawi, Lilongwe, Malawi; 3 Department of Obstetrics and Gynecology, School of Medicine, University of North Carolina, Chapel Hill, NC, United States of America; 4 Department of Health Behavior and Health Education, University of Michigan School of Public Health, Ann Arbor, MI, United States of America; 5 Department of Health Policy, University of Zambia School of Public Health, Lusaka, Zambia; International AIDS Vaccine Initiative, UNITED STATES

## Abstract

To eliminate mother-to-child transmission of HIV (EMTCT), scalable strategies to enhance antiretroviral adherence for both antiretroviral therapy (ART) and pre-exposure prophylaxis (PrEP) are needed as part of integrated HIV and maternal-child health services. We developed *Tonse Pamodzi* (“all of us together”), an adaptable intervention integrating biomedical and behavioral components to support HIV treatment and prevention. We describe our intervention development process, which comprised formative qualitative research, a review of the literature, and technical input from stakeholders representing the community, health systems, and policymakers. The resulting intervention, described herein, integrates patient-centered counseling and engagement of a patient-selected adherence supporter for pregnant and breastfeeding women initiating ART or PrEP. Patients receiving the intervention engage in Integrated Next Step Counseling (iNSC) sessions delivered by trained counselors to build and maintain adherence skills. Each patient also has the option of selecting an adherence supporter (partner, family member, or friend) who may participate in iNSC sessions and provide adherence support outside of these sessions. This flexible intervention is adaptable not only to ART or PrEP use, but also to the needs and preferences of each woman and the clinical context. If shown to be acceptable and feasible, the Tonse Pamodzi intervention may be an important tool in continuing efforts for EMTCT.

## Introduction

Over the past two decades, advances in the prevention of mother-to-child transmission of HIV (PMTCT) have transformed the landscape of pediatric HIV in sub-Saharan Africa [1–3]. Pregnant and breastfeeding women living with HIV now have access to efficacious regimens that can significantly reduce vertical and horizontal HIV transmission, and minimize morbidity and mortality [4, 5]. Despite these gains, challenges remain that have impeded efforts to virtually eliminate mother-to-child transmission of HIV [5–7]. For example, nearly 20% of women drop out of care in the first six months of treatment [8–13]. Among those retained in care, as many as 30–50% demonstrate suboptimal adherence [14–16]. If unaddressed, poor adherence to ART can lead to antiretroviral drug resistance, treatment failure, and HIV transmission [14, 17–19]. While evidence-based interventions have improved ART adherence during pregnancy and breastfeeding [11], there remains a need for resource-appropriate and scalable approaches. Without feasible, acceptable, effective, and sustainable ART adherence support strategies, local governments will not realize the full potential of their PMTCT investments.

As PMTCT programs have expanded, new HIV infections during pregnancy and breastfeeding—often undiagnosed—have emerged as a growing contributor to pediatric HIV cases [20]. Women face a high risk of HIV acquisition over the course of pregnancy and breastfeeding [21, 22], which in turn poses a transmission risk to the fetus or infant [23–28]. For women testing HIV-negative early in antenatal care, services typically end with post-test counseling. Few, if any, structured interventions are offered. For women at elevated HIV risk, the World Health Organization recommends oral pre-exposure prophylaxis (PrEP) in the form of daily tenofovir-emtricitabine [29–32]. Although PrEP is a safe and effective when taken consistently [24, 33–35], challenges to sustained PrEP adherence among women persist [36–39]. Pregnant and breastfeeding women experience unique barriers to PrEP adherence (including changes in lifestyle and sexual activity), yet there are no known evidenced-based strategies to support pregnant and breastfeeding women’s adherence to PrEP [40].

In order to eliminate mother-to-child HIV transmission, comprehensive PMTCT programs must expand their scope to include maternal HIV treatment *and* prevention using a “status-neutral” approach [41, 42]. Scalable and sustainable approaches to support adherence—regardless of the underlying antiretroviral regimen—are needed. The *Tonse Pamodzi* intervention combines biomedical and behavioral components to address this gap. We describe how this intervention was developed and explain its principal components. If shown to be acceptable and feasible, this adherence support intervention could further enhance the integration of HIV prevention, care, and treatment within antenatal care in diverse African settings.

## Materials and methods: Intervention development

The Tonse Pamodzi intervention is aligned with our overarching framework for HIV prevention during pregnancy and breastfeeding [43]. We posit that, in order to optimally reduce horizontal and vertical HIV transmission, strategies tailored to the HIV status of the patient and her partner(s) are needed. Although the armamentarium for HIV prevention is growing, antiretroviral medications remain foundational biomedical interventions, whether for HIV treatment (ART) or prevention (PrEP). To strengthen HIV treatment and prevention services for pregnant and breastfeeding women, we sought to develop a status-neutral intervention to enhance antiretroviral adherence through an adaptive and integrated approach. Our intervention development process included a formative qualitative study, stakeholder consultation, and identification of intervention components through expert consultation and literature review. We describe each of these activities below.

### Formative qualitative work

We conducted formative research to inform the design of our adherence support strategy. At sites in Lilongwe, Malawi and Lusaka, Zambia, we completed a total of 143 in-depth interviews with various groups, including pregnant and breastfeeding women, male partners, healthcare workers (HCWs), and policymakers. Several findings from these interviews informed the intervention. First, participants emphasized the important role of social support for medication adherence, especially from male partners. This desire was embedded in social and cultural norms, and was a key factor in women’s medical decision making. Second, among many participating women, there were formalized ways in which male partners provided adherence support. This included providing reminders about clinic appointments and, for women who were HIV-positive and on ART, supporting adherence and assisting with medication collection. Participants offered suggestions about how such engagement could be increased, including structured involvement in clinical care. Third, with the exception of policymakers, PrEP was not well-known to most groups. This was not surprising since PrEP had only recently been introduced (in Zambia) or in early policy discussion (in Malawi) at the time of the study. Nevertheless, patients viewed PrEP positively; many indicated that, if made available, they would be interested in initiating PrEP. However, a common concern was the need for strict adherence and how this would be maintained over time. HCWs and policymakers also emphasized the need for resources—and new approaches—to promote PrEP adherence at the clinic level. Complete qualitative results have been published elsewhere [34, 44].

### Ethical considerations

For the formative qualitative study above, we received ethical approval from the University of North Carolina at Chapel Hill Institutional Review Board (Chapel Hill, NC, USA), the National Health Science Research Committee of Malawi (Lilongwe, Malawi), and the University of Zambia Biomedical Research Ethics Committee (Lusaka, Zambia) to conduct this study. All participants provided written informed consent prior to initiating study activities.

### Technical consultation with stakeholders

We synthesized the formative findings alongside results from mathematical modeling and systematic reviews [22, 45, 46] to develop an HIV prevention package for pregnant and breastfeeding women. To facilitate this process, in September 2018, we convened a technical consultation with stakeholders representing the community, health systems, and policymakers. Participants included our study team, steering committee, and invited experts from the U.S., Malawi, Zambia, and South Africa. The objectives of this two-day meeting were to review ongoing research in HIV prevention during pregnancy and breastfeeding in Zambia and Malawi—including our formative work—and vet proposed interventions for our target population. Our panel identified potential obstacles to ART and PrEP adherence for pregnant and breastfeeding women, including barriers at the individual, relationship, and structural levels. These were based on the existing medical and public health literature. Intervention approaches, including patient-centered counseling and external social support for adherence, were identified which could address each barrier (Table 1). Additional key stakeholder recommendations for our intervention included: (1) adaptation of services to promote male engagement; (2) integration of new services within existing structures; (3) promotion of awareness for newly introduced prevention modalities, including PrEP, and (4) consideration of structural and behavioral barriers to antiretroviral drug adherence.

**Table 1:** Potential barriers to antiretroviral therapy and/or HIV pre-exposure prophylaxis adherence, as identified in a technical consultation meeting (September 2018), mapped to recommended intervention components.

	Patient-centered counseling	External adherence supporter
**Individual-level barriers**		
Lack of knowledge about ART or PrEP	•	•
Low perceived risk, feeling healthy	•	•
Fear of stigma	•	•
Conflicting demands and responsibilities	•	•
Traditional and spiritual beliefs	•	
Substance use	•	•
Mental health	•	•
**Relationship-level barriers**		
Power dynamics–social, cultural	•	•
Partner violence	•	•
Fear of disclosure (HIV status, PrEP use)	•	•
Poor social support	•	•
**Structural-level barriers**		
Poor treatment by healthcare providers	•	
Transportation	•	•
Discreet storage of medications	•	•
Food requirements for medication	•	
Clinic wait times	•	•
Quality of counseling	•	

### Identification of intervention approaches

From the formative study and stakeholder recommendations, a patient-centered adherence counseling approach emerged as a foundational component of the intervention. As motivational interviewing and other patient-centered counseling had been evaluated in the context of HIV prevention and treatment, we sought to adapt an evidence-based strategy to address ART and PrEP adherence. We consulted with experts in HIV adherence and reviewed counseling approaches familiar to the study team. Through this process we identified the Integrated Next Step Counseling (iNSC) approach for adaptation. This patient-centered approach had been used in the context of HIV; was found to be acceptable and appropriate for the target population; and retained sufficient flexibility to address challenges faced with both ART and PrEP [47, 48].

To foster external adherence support as part of our intervention, we conducted a literature review focusing on couples-based and other social support-based behavioral interventions to support adherence to ART or PrEP. Relevant interventions were identified through a systematic search of PubMed, Clinicaltrials.gov, and the Centers for Disease Control and Prevention (CDC) Evidence-Based Intervention database, and through content expert recommendation. The search yielded 17 unique interventions and revealed mixed evidence of effectiveness. Examples of diverse models for adherence support interventions spanned three basic categories of support: couples-based support for adherence; support from an individual selected by the patient (often a family member or friend); and support from a “peer” assigned by the study, often another patient. We identified the programs in Table 2 as those most salient for our intervention objectives and context, prioritizing interventions with one or more of the following characteristics: evidence of a treatment effect on adherence or intervention acceptability/feasibility; appropriateness to the African context; and potential for scalability and sustainability in low- and middle-income country settings. Common features that were incorporated into these interventions included the selection of an adherence supporter with an existing relationship with the patient and integration of individual counseling and dyadic support options.

**Table 2:** Prior adherence support interventions informing social support intervention component.

Intervention	Context	Intervention description	Adherence effect summary
COMDIS Treatment Supporter program [49]	Uganda	Close confidant selected by the participant and trained by staff to provide social support and directed adherence support.	Intervention participants had >4x the odds of achieving optimal adherence (OR = 4.51, p = 0.027].
Treatment Partner-Assisted Therapy [50]	Uganda	Treatment partners selected by patients trained to conduct directly observed therapy and support side effect management & prescription pickup.	Undetectable viral load at 24 weeks was achieved by 61.7% of intervention arm vs. 50.2% of control (OR = 1.58, p<0.05].
HEART program [51]	United States	Nurse-delivered counseling and education for both the patient and a support partner selected by the participant.	40% of intervention group adherent vs. 28% of control (p = .02)

### Development of intervention components

We sought to develop a unified approach to support both ART and PrEP adherence. Based on our formative work and the recommendations from the technical meeting, we adapted and developed two intervention components: (1) tailored patient-centered counseling focusing on adherence *and* sexual health and well-being, and (2) external adherence support. These strategies addressed many barriers identified in our technical consultation (Table 2) and could be tailored for either ART or PrEP.

To enhance facility-based adherence support, we adapted an existing counseling approach: Integrated Next Step Counseling (iNSC) [47, 48]. iNSC is a patient-centered, highly tailored approach to adherence support and has been used in previous clinical trials [47, 48]. It comprises nine key steps that engage the patient and ensure that discussions are interactive, targeted, and actionable. iNSC incorporates motivational interviewing techniques and draws upon the Information, Motivation, Behavioral Skills model [52, 53].

Given the prominence of external adherence support in our formative research and literature review, clinic-based iNSC was paired with an adherence supporter intervention. The adherence supporter component of our intervention draws upon common elements of relevant prior interventions identified in our literature review, including: selection of a supporter with an existing relationship with the patient; facilitating lay adherence support through a brief orientation; and involving the supporter in dyadic adherence support counseling sessions. This intervention component was informed by social support theories, which suggest that such support can bolster coping efforts [54], facilitate problem solving, and provide access to essential information and resources to support health behaviors including medication adherence [55, 56]. Specifically, we aimed to operationalize dimensions of social support described by House [57]: emotional support, instrumental support, and informational support.

We documented the content for each component of the Tonse Pamodzi intervention in an iteratively developed intervention manual. A core study team adapted existing iNSC manuals to address the needs of pregnant and breastfeeding women using PrEP or ART. This team also created the training and counseling content involving the external adherence supporter, including an orientation session for the supporter and joint iNSC sessions. Study team members met regularly to refine the intervention manual and sought input from study counselors in structured ways to address feasibility and acceptability.

After developing the initial intervention manual, changes were made based on feedback from study counselors and audits of early intervention sessions. During a three-day training, study counselors gave their feedback on the intervention manual and recommended that we focus on addressing the general wellbeing of women newly diagnosed with HIV during the first iNSC session. They also suggested that the discussion of adherence supporter selection follow the first iNSC session (rather than being integrated into the session itself). Finally, they proposed that the roles for iNSC counseling and adherence supporters be separated, to ensure the confidentiality of the information shared by the participant.

Following the training, the intervention development team demonstrated sessions and counselors piloted the intervention manual by role playing in pairs. These sessions were audio-recorded, transcribed, and translated to English. The intervention development team provided feedback on these transcripts as well as transcripts of the first two iNSC sessions conducted by each counselor. Through this process, we observed a tendency toward counselor-directed prescriptive education rather than participant-centered counselling as intended. As part of our ongoing training, case scenarios and role plays were conducted to improve counselling style and ensure fidelity to the intended the patient-centered nature of iNSC counselling. In early intervention sessions, some participants were confused by requests to make decisions to tailor their care (e.g., nomination of an adherence supporter). The counselling approach was adjusted to emphasize to women that they had agency to determine the course of the intervention, a paradigm shift from typical health care discussions in the study setting, but a shift which most women embraced. The intervention manual was finalized based on these recommendations and lessons learned. Below, we describe the content of the final intervention components, which are under evaluation in an ongoing pilot study. The full intervention manual is included as S1 Appendix.

## Results: Intervention description

**Fig 1:**
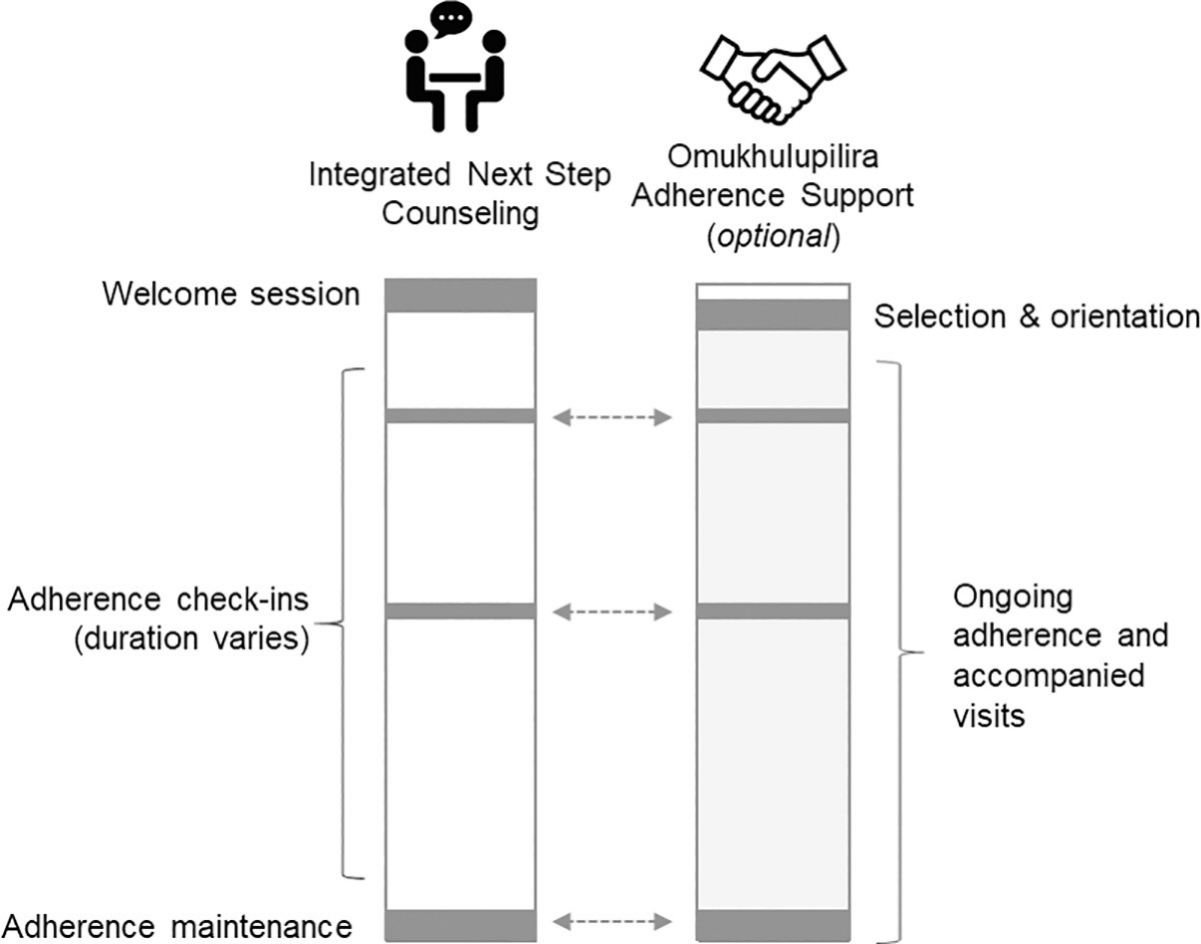
Tonse Pamodzi intervention overview: Enhanced ART & PrEP adherence support.

The Tonse Pamodzi (“all of us together” in Chichewa and Nyanja) program is an adaptable, integrated intervention to support antiretroviral adherence. It is an *adaptable* approach that can be used to support either ART or PrEP adherence based on the needs of each woman, as well as the clinical context. The intervention also comprises *integrated* biomedical and behavioral components. The intervention consists of two main components identified through formative research and stakeholder recommendations: iNSC and external adherence support (Fig 1). The iNSC counseling consists of regular one-on-one and optional dyadic counseling sessions to build and maintain adherence skills. The selection of an adherence supporter is optional but, if the patient elects, the supporter can participate in iNSC sessions and provide adherence support outside of these sessions. These intervention components are interdigitating, with flexible options to involve the adherence supporter in iNSC sessions, according to the patient’s preference. We describe the content of each intervention component below. Further details can be found in the intervention manual (see S1 Appendix).

### Integrated Next Step Counseling (iNSC)

The iNSC Counseling for the Tonse Pamodzi intervention consists of a conversation opened with an invitation to explore experiences and intentionally framed as a process rather than a structured discussion. The basis of the conversation is to understand the context in which each woman is experiencing her own sexual health. The iNSC discussion assumes that the woman is the expert of her experiences, that experiences are influenced by multiple factors, that there are diverse pathways to adherence and engagement in sexual health, and that facilitated exploration can lead a patient identifying her own adherence needs and strategies. Communication is intentionally neutral (i.e., non-judgmental), avoids telling women what they must or should do, and draws on strengths, resources, and facilitators. However, counselors influence the direction of the conversation moving through its component steps (Table 3) with probing, questions, and reflections. Women’s experiences navigating sexual health or HIV care can involve personal, interpersonal, social, community-based, or structural factors. Strategies and goals emerging from iNSC discussions can similarly span these levels and iNSC counselors are prepared with active referrals and the ability to link ART and PrEP users to wrap-around services.

**Table 3:** Overview of Integrated Next Step Counseling (iNSC) steps.

Step	Content
Introduce	Explain what you want to discuss, why, and ask permission
Frame discussion	Frame discussion as involving two topics (typically, first about general well-being and then about adherence). *Steps below will be repeated for each component*.
Review	Check in on previous goals/discussions, close and move into current experiences (follow-up visits only)
Explore	Explore the socio-ecological factors that optimize and that challenge the topic area being discussed (e.g., well-being, sexual health, PrEP/ART start decisions, PrEP/ART uptake, PrEP/ART adherence, or PrEP/ART adherence)
Tailor	Use the context and experiences shared to focus in on a specific aspect of the topic area (e.g., well-being, sexual health, PrEP/ART start decisions, PrEP/ART uptake, PrEP/ART adherence, or PrEP/ART adherence) the is relevant, meaningful and important to the participant to direct the focus of the next question and remaining discussion
Identify	Ask what would need^1^ to happen in any of the socio-ecological levels for the specific aspect of the topic area (identified above) to be easier to handle or be more manageable—what would need to happen for movement from her current situation to where she would like to be.
Strategize	Ask how the participant could see the need (identified above) being met
Agree	From list of ways the need could be met, ask the participant if she would be willing to try out one or more of those strategies before she returns to clinic again. Explore action plan and help participant to develop method of evaluating whether or not the strategy helped with the need and related next steps
Transition/ Close	Move to a new topic and repeat the flow OR close the discussion
**Joint session with adherence supporter (Omukhulupilira)**	For participants with an adherence supporter, they are first asked permission to invite their adherence supporter into the discussion, discuss exactly what aspects of their needs, strategies, and goals they are comfortable sharing in the joint discussion, and then the joint session includes an appropriate summary and exploration of the supporter’s thoughts and insights around the shared material. Tangible and emotional social support from the supporter, when offered, become part of the action plan.

*Omukhulupilira may be included in an abbreviated dyadic session (covering final 3 steps) following the main individual session if the participant desires

^1^ The participant’s *need* (eg., need for intimacy, support, connection, access, motivation) is distinct from actions or behaviors one would execute to meet a need. For example, one may go for HIV testing (strategy) to feel connected to and supported by partner (need). Thus, when participants respond to what they need with a strategy, counselors probe to understand what that strategy is doing for the person- what need is it helping to address.

The iNSC counseling builds upon the standard of care education that women receive regarding HIV when they initiate either ART or PrEP. In addition, the intervention can draw upon any existing adherence monitoring that is standard in the implementation context, including pill counts, self-report, electronic monitors, or pharmacologic monitoring. If patient-specific adherence information beyond self-report is available, this could be used in the iNSC adherence support discussions.

iNSC is conducted by trained counselors. Sessions vary in length but the first session is typically 30–45 minutes with follow-up sessions taking about 15–30 minutes. The general structure of each session follows the steps outlined in Table 3. The Tonse Pamodzi intervention includes three iNSC session types (Fig 1):

- An initial *Welcome Session* begins with a review of the standard of care education on HIV. This is followed by a general discussion focused on the challenges and facilitators of maintaining sexual health and well-being. Finally, this moves to a targeted discussion about how to achieve and maintain medication adherence.
- *Adherence Check-In Sessions* can be offered every 1–3 months for the desired duration of adherence support. These sessions begin with a review of the previous discussion and goals set in the last session. They then follow the two-part iNSC structure described in the Welcome Session—a broader discussion about general health issues followed by a conversation focused on antiretroviral adherence. Any available adherence monitoring information is documented in clinic notes that can be used by counselors during the session.
- A final *Adherence Maintenance Session* closes out the enhanced adherence support and facilitates transition to standard of care. The aim of this final session is to identify new barriers to medication adherence and how to address them. It is also used to summarize past strategies and provide each woman with some insights into continued engagement in this problem-solving approach. Following the two-part discussion about general well-being and adherence, the session closes with a discussion of future steps for continued adherence and successful transition to the standard of care.

### Omukhulupilira

Women receiving the Tonse Pamodzi intervention are given the opportunity to identify an adherence supporter or *Omukhulupilira* (“close confidant” in Chichewa and Nyanja). This individual is educated and trained to support the patient’s use of ART or PrEP, and may accompany her to clinic visits. The Omukhulupilira is a partner, family member, or friend chosen by the patient who can provide emotional, instrumental, and informational social support to help the patient adhere to her ART or PrEP regimen. Selecting this type of supporter is a recommended but not mandatory. The Omukhulupilira receives a brief in-person orientation session on how to provide positive support to the patient. At the patient’s invitation, they also may join portions of the iNSC sessions. Their role outside of these sessions will be determined by orientation session and iNSC discussions and according to the needs and preferences of the patient.

Near the close of the first iNSC session, the counselor introduces the role of the Omukhulupilira and works with the patient to determine whether she is ready to select an Omukhulupilira. The counselor helps the patient think through the suitability of potential Omukhulupilira candidates (e.g., if have they disclosed their HIV status to this person). If the patient chooses to nominate an Omukhulupilira, she is asked to invite them to attend a special orientation session. If the patient is not ready to select someone, she may select a supporter at any future visit.

The Omukhulupilira Orientation Session conducted by intervention counselors serves to educate the Omukhulupilira about ART or PrEP and their role in supporting the patient’s use of ART or PrEP, and to coach them in the best ways of providing this support. First, depending on the patient’s HIV status, the Omukhulupilira receives the local standard of care education on HIV and ART or PrEP. Second, they receive an explanation of their role as an Omukhulupilira, including their role both during and outside of iNSC sessions. The counselor also engages them in a discussion about barriers to ART or PrEP adherence to inform the importance of social support for adherence. Third is a participatory discussion about different types of social support and ways of providing them including: emotional support (expression of empathy, acceptance, love, trust, and care) including the principle of empathy and ways of providing empathetic verbal support; instrumental support (tangible aid and service); and informational support (advice, suggestions, and information). The principles of non-punitive support (encouraging adherence without expressing anger, violence, or withholding material needs) and patient-centeredness (putting the needs, perspective, and opinions of the patient ahead of their own) are also discussed. Finally, the counselor works with the Omukhulupilira and the patient to discuss specific support that the patient needs or desires, and to plan their first meeting outside of the clinic.

Following this orientation, the Omukhulupilira is equipped to provide ongoing support to the patient as needed. They may also participate in future iNSC sessions at the invitation of the patient. If invited, after the patient completes the primary individual iNSC session the Omukhulupilira may then join for an abbreviated joint session which is condensed to the final three steps of the iNSC framework (Table 3). In this joint session the Omukhulupilira is asked to help identify and develop strategies to address adherence barriers faced by the patient. Only the barriers which the patient wishes to share with the Omukhulupilira are discussed. The iNSC counselor encourages engagement of the Omukhulupilira and ensures that s/he is given the opportunity to share observations and experiences in a manner that fosters joint problem solving.

## Discussion

Our goal was to develop an integrated behavioral intervention that can be adapted to support ART or PrEP adherence during pregnancy and breastfeeding. Informed by the literature, formative research, and stakeholder engagement, we developed the two key components of the Tonse Pamodzi intervention: enhanced adherence counseling and facilitated adherence support. A core strength of this approach lies in its flexibility to meet the needs and preferences of each patient across multiple dimensions. First, it is a unified approach to support either ART or PrEP users, making it applicable regardless of why antiretroviral drugs are being used. It also complements rather than replaces standard of care adherence support, being flexible to build upon whatever adherence education and monitoring are locally available. With these attributes, the intervention could be integrated into existing PMTCT services to provide enhanced adherence support to new ART or PrEP initiators, or as targeted support for those facing specific adherence challenges. While the intervention can build upon standard of care antiretroviral adherence support, it goes far beyond the didactic and education-based approach which is typical of PMTCT adherence counseling in the intervention setting. Rather, the Tonse Pamodzi person-centered approach is built on the foundation of motivational interviewing techniques and social support, both of which have been used effectively in other contexts to promote medication adherence [55, 56, 58].

Second, the intervention provides adaptable facilitation of social support from a partner, family member, or friend, but only if desired by the patient and on her terms. It does not assume the patient has a supportive relationship with a male partner or would prefer to receive adherence support from him (though this may often be the case), but rather allows her to have anyone she desires as a supporter—or no supporter at all. We left this choice open to patients to ensure the relevance of the intervention for every woman, and to avoid partner-related social harms. Some women may not have close and supportive relationships with their male partners; instead, they may have more supportive relationships with members of their immediate or extended family [59]. Furthermore, while partner HIV status disclosure may be important for treatment engagement [60], and women taking PrEP may also benefit from disclosing their PrEP use [61, 62], some may wish to avoid PrEP use disclosure. For example, women experiencing intimate partner violence (which is associated with HIV risk [63]), may fear inciting violence if their partner learns of their PrEP use [64].

Finally, the intervention is adaptable to the desired duration or period of enhanced adherence support, though the immediate and sustained effectiveness of offering the intervention at different periods and durations needs to be evaluated. In describing the intervention, we have purposely not defined the intended duration of the iNSC counseling and facilitated adherence support as both could be continued as long as they are useful to the patient and feasible in the implementation context. We are currently evaluating the Tonse Pamodzi intervention delivered over six months to assess the implementation and preliminary effectiveness of the intervention in Lilongwe, Malawi (NCT04330989). Through two randomized pilot trials (for women initiating ART and PrEP, respectively), we will assess intervention acceptability, fidelity, and clinical outcomes associated with the Tonse Pamodzi intervention using a mixed-methods approach. We will also assess the preliminary effectiveness of the intervention on retention in care and medication adherence over six months. For these trials, we chose to deliver the intervention over six months following ART or PrEP initiation, as this is a critical period for establishing adherence skills and overcoming challenges [11–13, 65]. However, in practice the counseling could be extended or shortened as desired. Data from these ongoing studies will provide insights about the intervention’s acceptability and feasibility, which are to date unknown. Future work is also needed to understand its scalability and sustainability, particularly if used as part of a public health approach to HIV prevention, care, and treatment.

## Conclusions

The Tonse Pamodzi intervention is a unified and adaptable model of enhanced antiretroviral adherence support for pregnant and breastfeeding women using ART or PrEP. These components were adapted and developed through formative research and stakeholder consultation. We are currently evaluating the acceptability and feasibility of the Tonse Pamodzi intervention for retention in care and medication adherence in a pair of randomized pilot trials in Lilongwe, Malawi. If shown to be promising, this intervention may be an important tool in continuing efforts to eliminate mother-to-child HIV transmission.

## Supporting information

S1 Appendix Tonse Pamodzi adherence support intervention manual. (PDF)
