# Supplementary material for: Tonse Pamodzi: Developing a combination strategy to support adherence to antiretroviral therapy and HIV pre-exposure prophylaxis during pregnancy and breastfeeding
Source: PLoS One. 2021 Jun 25;16(6):e0253280. doi: 10.1371/journal.pone.0253280 (PMC8232532; doi:10.1371/journal.pone.0253280)
Supplement: S1 Appendix — (PDF) [file pone.0253280.s001.pdf]

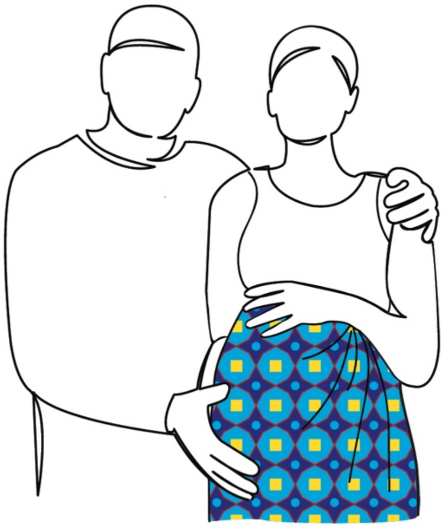

TONSEPAMODZI

ADHERENCE SUPPORT INTERVENTION MANUAL

Contents

ACKNOWLEDGEMENTS 2
INTRODUCTION 3
INTERVENTION COMPONENTS 3
1. Integrated Next Step Counselling (iNSC) 3
2. Adherence supporter model (Omukhulupilira) 3
OVERVIEW OF INTERVENTION SESSIONS 4
FACILITATOR/COUNSELOR PREPERATION 4
ENROLLMENT VISIT 6
SESSION COMPONENTS 6
1. WELCOME AND OVERVIEW 6
2. iNSC 7
3. EXPLORATION AND SELECTION OF OMUKHULUPILIRA OPTION 8
4. CLOSE THE SESSION WITH PLAN FOR NEXT STEPS 10
OMUKHULUPILIRA INVITATION CARD (SAMPLE) 13
OMUKHLUPILIRA NOMINATION FORM (SAMPLE) 14
OMUKHULUPILIRA ORIENTATION VISIT 15
1. INTRODUCING THE ORIENTATION VISIT 15
2. EDUCATION ABOUT HIV AND ART/PREP 17
3. THE ROLE OF THE OMUKHULUPILIRA 17
4. WAYS OF PROVIDING SUPPORT FOR PREP/ART ADHERENCE 18
5. FACILITATING INITIATION OF SUPPORT 20
6. CLOSING THE SESSION 22
ORIENTATION VISIT CHECKLIST 23
ADHERENCE CHECK-IN VISITS (MONTHS 1 & 3) 24
SESSION COMPONENTS 24
1. WELCOME 24
2. iNSC 24
3. JOINT SESSION WITH OMUKHULUPILIRA 25
4. CLOSE THE SESSION WITH PLAN FOR NEXT STEPS 25
ADHERENCE MAINTENANCE VISIT (MONTH 6) 26
SESSION COMPONENTS 26
1. WELCOME 26
2. iNSC 26
3. JOINT SESSION WITH OMUKHULUPILIRA 27
4. CLOSE SESSION WITH PLAN FOR NEXT STEPS 27
APPENDIX I. iNSC WORKBOOK FOR CLINICAL RESEARCH SITES
APPENDIX II. EDUCATIONAL MATERIALS

Acknowledgements

We would like to acknowledge the following individuals for their roles in creating this training manual: Friday Saidi, Lauren Hill, Benjamin Chi, Kellie Freeborn, Nora Rosenberg, Suzanne Maman, and Wilbroad Mutale. We would also like to thank K. Rivet Amico for her valuable guidance and many reviews of the manual. The development of the Tonse Pamodzi adherence support intervention was funded by the National Institutes of Health (R01AI131060).

This training manual was developed using formative data from the Tonse Pamodzi study; input from the co-investigators listed above; and training materials for Integrated Next Step Counseling, an approach developed by Amico, et al. for the iPrEx Study ("Iniciativa Profilaxis Pre-Exposición"). We have adapted these materials to fit the Malawian and Zambian settings, as part of a broader adherence support package that includes self-selected adherence supporters.

INTRODUCTION

If the anticipated gains for the biomedical prevention of mother-to-child HIV transmission (PMTCT) programs are to be realized, adherence to antiretroviral regimens for both treatment and prevention of HIV are critical. Adherence can be defined as the extent to which a patient follows a treatment plan, takes prescribed medicines as directed, and follows any related restrictions regarding food and other medications. There are other important factors that go into successful outcomes—like coming in for recommended care or medication refills (retention in care), the length of time someone is committed to a given treatment (persistence), or a mix of all of these (patient engagement).

Over the years, different models and frameworks for how and why people may struggle with adherence particularly have been developed and so have different approaches to trying to optimize outcomes for antiretroviral therapy (ART) and pre-exposure prophylaxis (PrEP). Humans are ultimately social creatures, so it is not surprising that partner, friend, and/or family support can be very valuable for women and improve adherence and retention in antenatal and HIV programs. Adherence support from team members trained in patient-centered, person-facing counselling and communication can also help individuals to optimize medication adherence.

This workbook describes an intervention that is a combination approach to improve adherence for women initiating antiretroviral therapy (ART) or pre-exposure prophylaxis (PrEP) during pregnancy. It has been designed for the Tonse Pamodzi 2 study (TP-2). This combination intervention includes Integrated Next Step Counselling (iNSC) and an adherence supporter model (Omukhulupilira) that helps pregnant and breastfeeding women to develop their own strategies to improve adherence in the context of their overall well-being.

Women receiving this combination intervention will have dedicated time with a specialized counselor at their enrollment, Month 1, Month 3 and Month 6 study visits. In addition, they will be given the opportunity to identify an *omukhulupilira* ("close confidant" in Chichewa and Nyanja), an individual who will be educated and trained to support the participant's use of ART or PrEP, and may accompany the participant to study visits. Over the course of the four study visits, study counselors will provide iNSC and—if a support person is identified—support omukhulupilira interactions, in ways that encourage adherence to antiretroviral medications in the context of general well-being. This is described in greater detail in this manual.

INTERVENTION COMPONENTS

1. Integrated Next Step Counselling (iNSC)

iNSC is a two-phase discussion opened with an invitation to explore experiences and intentionally framed as a process rather than a series or set of messages. Although steps are articulated to guide implementers through the iNSC process, the driving goal is to engage participants in a non-judgmental discussion of their experiences surrounding protection of sexual health. iNSC is a process for having a conversation. It draws from the Information, Motivation, Behavioral Skills model situated within a socio-ecological context. The iNSC discussion assumes that the participant is the expert of their own experiences, that experiences are influenced by multiple factors, that there are diverse pathways to adherence and engagement in sexual health, that these pathways can be identified through exploration, and that facilitated exploration can lead to clients identifying their own needs and strategies. iNSC training emphasizes the importance of engaging in a genuine, as opposed to formulaic or predetermined, conversation framing the discussion around the specific context and needs of the participant. Communication is intentionally neutral (non-judgmental), avoids telling participants what they must or should do, and draws on strengths, resources, and facilitators.

2. Adherence supporter model (Omukhulupilira)

A recommended but optional component of the intervention, the omukhulupilira, will be a person chosen by each participant who can provide emotional, instrumental, and informational social support to help the participant adhere to their ART/PrEP regimen. In Chichewa and Nyanja, "omukhulupilira" means a close confidant. If and once selected by the participant, the omukhulupilira will receive a brief in-person training on how to provide positive support to the participant during a structured orientation visit. At the participant's

invitation, they will have the opportunity to join portions of the iNSC sessions after they have completed the orientation visit. In these sessions, the counselor and participant may call on the omukhulupilira to help discuss barriers to adherence, strategies to overcome these barriers, and their role in helping the participant to implement these strategies as appropriate. The role of the omukhulupilira outside of these sessions will be determined by this discussion and the needs and preferences of the participant.

OVERVIEW OF INTERVENTION SESSIONS

Visit	Participant activities	Omukhulupilira activities
Welcome Orientation (Day 0)	I. Welcome & education debrief<br>II. iNSC<br>• Health in context: sexual health (PrEP) or general well-being (ART)<br>• PrEP/ART adherence<br>III. Exploration & selection of omukhulupilira<br>IV. Close session	N/A not yet selected
Omukhulupilira orientation (As early as possible, but not restricted)	Encouraged to attend, but not mandatory	I. Education about HIV and PrEP/ART<br>II. Social support for adherence<br>III. Coaching to provide support<br>IV. Closing the session & next steps
Adherence check-in (Month 1 and Month 3)	I. iNSC session<br>• Health in context: sexual health (PrEP) or general well-being (ART)<br>• PrEP/ART adherence<br>*If the omukhulupilira is present, determine whether the participant would like to discuss further in a joint session*	If present, participate in discussion of selected adherence strategies and potential role to support these strategies
Maintaining adherence (Month 6)	I. iNSC session<br>• Health in context: sexual health (PrEP) or general well-being (ART)<br>• PrEP/ART adherence<br>• Discussion of future steps, including links to the standard of care<br>*If the omukhulupilira is present, determine whether the participant would like to discuss further in a joint session*	If present, participate in discussion of selected adherence strategies and potential role to support these strategies

FACILITATOR/COUNSELOR PREPERATION

All counselors must have completed training on procedures and received confirmation from the supervisory team that he/she is ready to implement the intervention. Additionally, counselors should not only be facile with the intervention approach (iNSC) but should also have had the opportunity to gain knowledge and feel confident in any of the education material that the participant will have received prior to the iNSC session (in case there are questions and to identify potential misinformation). Finally, awareness of crisis counseling techniques for those working with newly diagnosed pregnant women is required. Ongoing supervision and training are provided throughout the study by the supervisory team.

NOTES ABOUT THIS MANUAL

An important goal of this intervention is to learn from the participant and tailor counseling messages in their individual context. As such, it is critical that the conversation flows freely and is directed to the participant's own experiences, needs, and obstacles. In this manual, we include sample text in blue italicized font. These examples are meant as guides only and *should not* be recited verbatim.

ENROLLMENT VISIT

After consent and enrollment, participants will be randomized to intervention or standard of care. ALL participants will have engaged in [ART/PrEP] education before meeting with the intervention counselor, as required by the study protocol. As such, participants may feel tired, overwhelmed or otherwise distracted by the time she meets with the intervention counselor. We will deliberately include comfort check-ins and efforts to promote participant engagement in intervention conversations. Welcoming participants and providing an overview of the session, while checking in on their current state of mind, is essential to this and must be attempted before getting into exploration of sensitive topics like sex and health. This is particularly critical for women who only recently learned of their HIV status.

SESSION COMPONENTS

Participants assigned to the intervention condition will go from receiving the standard education information about [ART/PrEP] and clinic visits to meeting with trained intervention counselors. During this part of the visit, participants will be asked to engage in the following 4 general phases of interaction:

1. Welcome and education debrief
2. iNSC
3. Exploration and selection of omukhulupilira
4. Close session with plan for next steps

1. WELCOME AND OVERVIEW

The enrollment session begins with an overview of the intervention package.

Introducing yourself, the intervention and this session

My name is ___________. Thank you for meeting with me today! Thank you joining the study. You have been selected to take part in the study intervention, which will help you with [ART/PrEP]. I will be checking with you each time you come to clinic to see how things are going. For this first conversation together, I am hoping to spend some time getting to know you better, share information with you and explore your thoughts, feelings and plans for your journey with [ART/PrEP].

Check in on comfort

Before we begin, I want to check in with you. You have been here a while today [and for newly diagnosed "and no doubt this has been challenging"]. We will spend about 30-45 minutes talking together today. Before we begin, do you need to use the rest room or take a break? Can I get you a snack or beverage? [list whatever amenities you have at the site]

Explain the program and your role

This program works with people starting [ART/PrEP] with the goal of helping people to feel confident and motivated to follow their regimen to the best of their ability. Moreover, it gives people the space to talk frankly about their experiences, even when those experiences may involve challenges- like missing doses or just not feeling like taking doses. Our conversations are about your needs, and we do not share this information with your medical care providers. It is private. The only thing I cannot keep private are situations where you may be in danger. If that is the case, then you and I will work together with other members of the care team here to ensure your safety. Sound OK?

Thank you. Each time you come in for [ART/PrEP], I will be checking with you to see how things are going. For this first conversation together, I am hoping to spend some time getting to know you better, share information with you and explore your thoughts, feelings and plans for your journey with [ART/PrEP].

Ask permission to carry on

Does that sound OK to you? Do you have any questions before we begin?

Education debrief – before moving further, check in on information the participant received already today

You have received a lot of information from the clinic staff today. What did you find most meaningful in all that was shared?

[Reflect]

Are there any pieces of information you heard that were not so clear or seemed hard to understand or believe?

[Process]

Any time you have questions about the information you receive, please let me or other members of the study staff know. We can help to clarify or explain further. The more informed people are, the better!

2. iNSC

The iNSC component of the session includes two parts.

The first part is a broader discussion about general well-being. This initial phase provides some information about the situational context for adherence behaviors and helps to build rapport with the participant. For individuals on ART, the emphasis is on general well-being; for individuals on PrEP, the focus is on sexual health.

The second part focuses in on use of [ART/PrEP], including challenges and facilitators of adherence. This is a direct discussion about how to achieve and maintain medication adherence.

All intervention counselors will be trained in the conduct of iNSC, using the iNSC Workbook (see Appendix). The iNSC discussion structure is shown in the table below. This follows with usual steps of iNSC, with the exception of the *Review* step, which may be skipped in first sessions because there are no previous goals or iNSC discussions to review with the participant yet.

Step	Description
Introduce	Explain what you want to discuss, why, and ask permission
Frame discussion	Frame discussion to two components, first about general well-being and then about adherence. *Steps below will be repeated for each component.*
Review	Check in on previous goals/discussions, close and move into current experiences (follow-up visits only)
Explore	Discuss socio-ecological factors that challenge or could optimize a specific behavior
Tailor	Reflect on context and experiences shared to tailor remainder of the discussion
Identify	Ask what would be needed to happen for the behavior (identified above) to be easier to handle or be more manageable
Strategize	Ask how the participant might consider addressing this need
Agree	Ask the participant if she would agree to try out one or more strategies to address the identified need
Transition / close	Move to a new topic and repeat the flow OR close the discussion

3. EXPLORATION AND SELECTION OF OMUKHULUPILIRA OPTION

The intervention counselor introduces the role of the omukhulupilira and how he/she may help to support ART/PrEP adherence. Below is an example of how this discussion may start.

> *Part of this program is to talk together when you come in for your [ART/PrEP]. Another service that we offer involves what we call an omukhulupilira, someone outside of the clinic who supports you and helps you to take [ART/PrEP]. Being able to take a medication every day can be hard. Many people find it helpful to have a family member or friend that they can rely on to help them remember to take their medicine, attend medical appointments, keep their spirits up, or just be there to talk about things. Research suggests that people who have someone important to them help out with adherence and their efforts to [treat/prevent] HIV may have better chances of being able to do so.*
>
> *For this study, we recommend that participants select an omukhulupilira. This may be a partner, family member, or friend who can support your adherence both during and outside of study counseling visits. Their exact role will be determined by your preferences and needs. You also do not have to select an omukhulupilira if you do not want to. You can select someone today, or at any other study visit.*
>
> *If you desire, your omukhulupilira can attend study counseling sessions with you. If you wish, your omukhulupilira can join us at the end of the counseling session to discuss ways to support your adherence. Outside of the counseling sessions, she/he can help you to implement these adherence strategies and may be a helping hand or someone to lean on for emotional support. The exact support they provide will depend on your needs, but some things that are often helpful include:*
>
> - *Helping you remember to take your medication*
> - *Helping you get to the clinic or refill prescriptions*
> - *Providing encouragement or emotional support*
> - *Providing child care so you can attend clinic appointments*
>
> *Some people have important others in their lives that they want to involve in their efforts to use [ART/PrEP] as recommended. We also know that not everyone has or wants that. If you do have someone you could see being your supporter, we are able to work them into parts of our sessions so that your plans, goals or strategies can be supported by that person.*
>
> *Just knowing what you know right now, and how you feel right now, what is your reaction to/thoughts about an omukhulupilira?*

If the participant <u>is uninterested</u> in identifying an *omukhulupilira*, thank her and ask for permission to revisit this topic at the next session and skip to Part 4 (Close the Session) below.

> *Thank you. I understand that an omukhulupilira is not for everyone. When you think about it, what are the main reasons you do not want that right now? Thank you. I respect your careful consideration on this. I will check in with you at your next visit about this service—and periodically throughout the study—just in case something changes. Is this okay?*

<u>If the participant is interested</u> in selecting an omukhulupilira (or exploring further), cover the remaining sections below as appropriate.

Material Needed: the Omukhulupilira Orientation Visit Invitation Card, the Omukhulupilira Agreement Form.

A. Important considerations when selecting an omukhulupilira

> *As we've discussed, the support that you will receive from the omukhulupilira will depend upon your needs, your relationship with them, and their capacity to provide support. It will be slightly different for each person. There are few things to consider when deciding on the appropriate supporter for you:*

1) *In most cases, it will be helpful if the omukhulupilira is someone you can see regularly outside of the counseling sessions so they can provide the support that you agree upon together.*
2) *If you have not done so already, you will need to disclose your [if applicable: HIV status] and [ART/PrEP] use to the person you select as your omukhulupilira (if you choose one).*
3) *Once you have selected an omukhulupilira, we will ask them to come in for an orientation session so that we can coach them on how to support you outside of study counseling sessions. Your [if applicable: HIV status] and [ART/PrEP] use will be discussed during this orientation session.*
4) *Omukhulupilira may come with you to our clinic counseling sessions, but this is entirely up to you. If they come, they can join us after the counseling session to discuss how they can support your adherence.*

Is there someone you can think of—a friend or family member you trust, [if applicable: who knows your HIV status], and who you see often—who might be appropriate and willing to be your omukhulupilira?

Allow person time to think and respond.

If not ready, let participant know that you can check in again next time they come in or they can simply bring the person with them to the next visit. Then go to Part 4 (Close the Session).

If the participant remains interested, continue to the next section.

B. Explore possible candidates

Work with the participant to reflect on the possible advantages and disadvantages of approaching potential people for this role. Gauge their interest and comfort in approaching each possible candidate.

- *Are you currently staying with [candidate's name]?*
- *Do you feel comfortable informing [candidate's name] that you have started [ART/PrEP]? What will you tell them?*
- *Can you ask [candidate's name] to come in for an orientation visit? What words might you use?*
- *Do you see any problem(s) having [candidate's name] come here for the next visit? If yes, how so?*
- *Would you like [candidate's name] to join us after the counseling sessions to talk about how they can support your adherence?*

Allow participant to continue exploring advantages and disadvantages—and different considerations—for each of the potential omukhulupilira candidates. When the conversation feels ready to move forward, continue to the next section.

C. Nominating an omukhulupilira

Ask the participant if she feels ready to identify an omukhulupilira. If no, remind participant you will check in again or if she does identify someone between visits, she can just let you know or bring in the person to her next visit. Then move to Part 4 (Close the Session).

For participants who are ready to nominate an omukhulupilira, review and complete the Omukhulupilira Nomination Form with them and ask them to sign and date the form. If the participant is unable to sign their name, ask them to provide a fingerprint instead. Read the form aloud to the participant and help them to complete and sign the form

On this form we will record identification information about the omukhulupilira you named, and any alternate individuals who might participate if that person is not available. This information will help us to make sure that we have the right person in the case that you are not able to come to the Orientation Visit. We also want to make sure that you understand what will be discussed with the omukhulupilira if they attend this visit.

D. Scheduling a visit with the omukhulupilira

If the participant is able to attend the visit, find a day and time for the visit that works for them, and complete the invitation card accordingly, otherwise simply provide the card. Instruct them to provide the card to the person they have determined is able to serve as their omukhulupilira. If they have nominated multiple potential omukhulupilira, ask them how they will determine which person to provide the invitation card to. Thank the participant before transitioning to closing the visit.

Now that you've selected a potential omukhulupilira, he/she will need to come in to this clinic for an orientation visit to learn how to work with you and the counselor to support your use of [ART/PrEP]. While not mandatory, we encourage you to take part in this orientation visit along with the omukhulupilira. Do you think you will be able to attend the visit with your omukhulupilira?

4. CLOSE THE SESSION WITH PLAN FOR NEXT STEPS

Provide a summary of what was discussed, thank the participant and remind her of the next visit.

We have covered a lot today. Thank you for sharing with me. Before you go, I just want to summarize some important parts of our conversation. OK? You mentioned that you need ___________ to feel [well, motivated to protect yourself, committed to your health and wellbeing, to feel using condoms works, so on] and ___________ would be a strategy you would be willing to try out to help with that. And for [ART/PrEP], you need ___________ and you are going to try [strategy] to see if that can be addressed.

You also [identified/decided against] selecting an omukhulupilira at this time. [If applicable] We will schedule a time to meet with this individual, as previously described.

Thank you for talking with me. I look forward to asking you about how things went. It is OK to give something a try and feel it is not quite right. Something that sounds perfect here may feel not so perfect once you leave this space and get into your daily life. If that happens, keep trying. Keep thinking of ways you might work with [repeat needs] just like we did here together. Thank you!

iNSC CHECKLIST – EXAMPLE

STANDARD iNSC Tracking Forms (2 pages)

ID:

Visit Date:	Completed by:

PrEP/ART Status (as of today's visit	Support Discussion
☐ on or getting PrEP ⟶	Full iNSC
☐ on or getting ART⟶	Full iNSC

Sexual Health Promotion/Wellbeing Counseling – iNSC

1 INTRODUCE / FRAME: Introduction to session provided? ☐ yes ☐ no

2 REVIEW: The participant's experiences/goals reviewed ☐ yes ☐ no ☐ NA (first visit, no goals from last visit)

3 EXPLORE: Experiences with sexual health protection through behavioral strategies. What strategies are used/considered? What promotes those (makes it easy)? What challenges use of protection strategies (makes it hard)?

Facilitators (said by participant)	Challenges (said by participant)
☐being well informed	☐not feeling well informed
☐partner(s) supports strategies	☐partner(s) unwilling/reluctant/against to practice strategies
☐personal commitment (motivation) to staying HIV negative	☐ fearful of rejection or missed opportunity (ruining the mood)
☐confidence in negotiating strategies with sexual partner(s)	☐specific incentives to not use strategies (pay or trade)
☐having intimacy (closeness) with partner	☐not thinking that getting HIV would be bad
☐fits well into what I do sexually	☐thinking partners are HIV-negative without really knowing their status
☐feeling "at risk"	☐feeling down/sad (not caring about protecting self)
☐**none could be identified**	☐interferes with intimacy
☐other, specify:	☐drug or alcohol use (making decision making difficult)
	☐caught up in the moment
	☐**none could be identified**
	☐other, specify:

4 TAILOR: Level of engagement in this part of counseling: ☐low ☐medium ☐high

**5 IDENTIFY** or **confirm** needs: **Needs** (What would make using protection strategies or sustaining ones that work "easier"? Select all)	
☐feel better informed	☐have better concrete skills around negotiating strategies with partners
☐have access to strategies (condoms, HIV testing, lube)	☐gain partner support
☐be assertive/confident	☐basic living needs met (housing, food, safety)
☐have strategies that are sexy/fit into sexual life	☐**none could be identified**
☐feel more motivated	☐other, specify:

6 STRATEGIZE: How to meet needs discussed? ☐ yes ☐ no

Sexual Health / Well-being Goal:

7 AGREE on: Strategy and Action Plan? ☐ yes ☐ no

Medication adherence – iNSC

3 EXPLORE: Experiences with adherence. What has made adherence manageable/well integrated (PrEP easy)? What has challenged consistent PrEP use?

**Facilitators (said by participant)**	**Challenges (said by participant)**
☐match with routine/events	☐partying/drugs/alcohol
☐mobile/carry tools (e.g., pill box)	☐medication (too big, tastes bad)
☐personal commitment (motivation) to staying HIV negative	☐disruption in routine
☐memory aids/tools (e.g., alarm, calendar)	☐lack privacy
☐access	☐scared others will think HIV+
☐social support (partner(s), family)	☐side effects
☐feeling "at risk"	☐feeling down/sad (not caring about protecting self)
☐**none could be identified**	☐feeling not at risk
☐other, specify:	☐memory or organization problems
	☐Partner or family member taking PrEP away
	☐PrEP being stolen
	☐**none could be identified**
	☐other, specify:

4 TAILOR: Level of engagement in this part of counseling: ☐low ☐medium ☐high

**5 IDENTIFY** or **confirm** needs: **Needs** (What would make PrEP "easier"/manageable? What do current successful strategies DO for participant- what **need** do they meet? Select all)	
☐more information	☐manage side effects
☐have access to PrEP when needed	☐social support
☐remember dose times	☐basic living needs met (housing, food, safety)
☐motivation (to feel like taking it, positive reasons to take it)	☐**none could be identified**
☐have privacy	☐other, specify:

6 STRATEGIZE: Strategies discussed? ☐ yes ☐ no

7 AGREE on: Strategy and Action Plan ☐ yes ☐ n

Adherence Goal:

OMUKHULUPILIRA INVITATION CARD (SAMPLE)

Invitation

Dear ______________________________:

At [Clinic Name] we are providing integrated services for pregnant women involving their partners, family members, or other loved ones who are supporting them in their pregnancy. We ask you to visit the antenatal clinic so that we can provide you with important information to ensure the best care for your partner, family member, or friend.

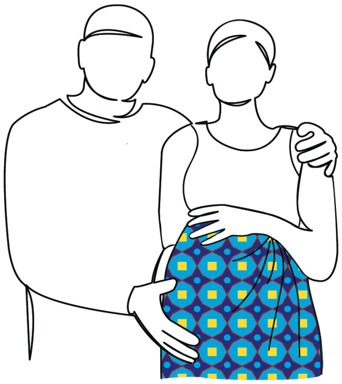

TONSEPAMODZI

Date: ___________________Time: ______________
Room: ____________________

You may come on another day (Mon to Fri)/time ([CLINIC HOURS]).

Bring this card and you will be attended to right away.

INVITATION #: ____________

OMUKHLUPILIRA NOMINATION FORM (SAMPLE)

TRIAL 1 PARTICIPANTS:
I, ____________________________, wish to nominate an individual to serve as my omukhulupilira, to help support my adherence to medicines for HIV treatment. I understand that, in order to serve in this role, the omukhulupilira will need to know my HIV status and that I am currently using antiretroviral medicines.

TRIAL 2 PARTICIPANTS:
I, ____________________________, wish to nominate an individual to serve as my omukhulupilira, to help support my adherence to medicines for HIV prevention. I understand that, in order to serve in this role, the omukhulupilira will need to know that I am currently using pre-exposure prophylaxis (PrEP) for HIV prevention.

I expect to invite the following person as my omukhulupilira, who I will ask to attend the Omukhulupilira Orientation Session:

**ID information for omukhulupilira**	
Name/Nicknames	
Relationship to participant	
Gender	
Age (approximate)	
Brief physical description (optional)	
Phone number (optional)	

If this person is unavailable, I may send the following alternate person(s) to attend:

**ID information for omukhulupilira: alternate 1**	
Name/Nicknames	
Relationship to participant	
Gender	
Age (approximate)	
Brief physical description (optional)	
Phone number (optional)	

**ID information for omukhulupilira: alternate 2**	
Name/Nicknames	
Relationship to participant	
Gender	
Age (approximate)	
Brief physical description (optional)	
Phone number (optional)	

____________________________________ ____________________

PARTICIPANT SIGNATURE OR FINGERPRINT DATE

INVITATION # ISSUED: ________________

OMUKHULUPILIRA ORIENTATION VISIT

Lay support for each participant's adherence to ART or PrEP is an integral component of the Tonse Pamodzi intervention. Social support from an important person in the participant's life can greatly reinforce their own individual efforts to take their medication by the prescribed schedule. The omukhulupilira is a partner, family member, or friend selected by the participant to support their adherence to ART/PrEP outside of study visits. They may also take part in adherence strategy-building following iNSC sessions at the clinic, depending on the participant's preference. Prior to this visit, the participant will nominate someone to attend this orientation visit (see Section 3 in Enrollment Visit section).

To help the omukhulupilira in this role, this orientation session is designed to educate them about ART/PrEP and their role in supporting the participant's use of ART/PrEP, and to coach them in the best ways of providing this support. This section includes the orientation visit procedures and the orientation visit checklist

The scripts provided below are examples only. The Orientation Visit Checklist and the corresponding headers provided in the text indicate the necessary components of the session.

1. INTRODUCING THE ORIENTATION VISIT

Check in and build rapport

[Begin by checking in and building rapport with the person. Then introduce yourself and the session].

Confirm omukhulupilira identity (if participant not present)

The participant is encouraged to accompany the omukhulupilira to the site for this orientation. This is to reduce problems with proper identification and issues around HIV status or ART/PrEP use disclosure. However, if they were unable to accompany the omukhulupilira, be sure to begin by verifying the omukhulupilira's identity using the instructions below.

If the participant *is not* present, ask the person to state the name of the participant who sent them, their name, and their relationship to the participant. Verify this information as well as their physical description, approximate age, and gender against the omukhulupilira ID information provided by the participant. Only proceed with the visit if you are able to verify their identity as one of the possible supporters named by the participant.

If the participant *is* present, you do not need to verify the identity of the omukhulupilira as above, but check with the participant first that the person they have brought with them is the individual they want to be their omukhulupilira. This can be done privately when the participant first arrives.

Once the person is confirmed by the participant as the intended omukhulupilira, record the omukhulupilira's gender and relationship to the participant in the appropriate fields of the Orientation Visit Checklist.

Confirm disclosure of HIV status (if applicable) and ART/PrEP use

Ascertain if participant has disclosed HIV status (if applicable) and ART/PrEP use to the supporter. Be careful not to disclose any information about HIV status or medications on behalf of the participant.

If participant is present, in private conversation to confirm omukhulupilira selection:

- Ask if she has already disclosed her [if applicable] HIV status and ART/PrEP use with the omukhulupilira.
- If she HAS NOT disclosed, make sure the woman is aware the counselor cannot disclose on her behalf

I do not have the right to disclose your HIV status or ART/PrEP use, but I can help to guide the conversation to help you disclose. Do you want to disclose in my presence?

If participant *is not* present, ask the person to explain what they understand about the purpose of their visit to the clinic. Probe on whether support for health or medication was mentioned. If disclosure has not occurred, conduct brief discussion of pregnancy support and close session (see below).

Would you tell me what [Name of Participant] told you about the purpose of this visit today?

If the participant has not disclosed and is either not present or does not wish to disclose the above, explain to the prospective omukhulupilira that this is a check in to see how the pregnancy is going. Engage them in a discussion regarding:

- Their understanding of issues the participant may be facing in their pregnancy
- If there is any way that they can help to support the participant in their pregnancy
- If there is anything the clinic can do to better support the participant in their pregnancy

Provide session overview

When HIV status and/or ART/PrEP use disclosure is confirmed, proceed:

So, as we've discussed, [Name of Participant] asked you to support her in taking [ART/PrEP]. We are calling people like you in this role "omukhulupilira."

Would you tell me a little bit about your relationship with [Name of Participant]?

How do you feel about being asked to play this role and help [Name of Participant] in this way?

Thank you for sharing. It's important to me to know your questions and concerns about being an omukhulupilira for your [family member/partner/friend], and we will take the time to address these issues over the course of our conversation today. We'll begin the session by providing you with information about [ART/PrEP], and why taking it every day as prescribed is so important. Then we'll talk about what is expected of you as an omukhulupilira, and talk about the ways that you can best support [Name of Participant]. Finally, we'll talk about what happens next after this visit, and the steps you can take between this visit and the next to support [Name of Participant].

Before we begin, are there any questions you have for me?

Discuss importance of confidentiality

Ask supporter to agree to confidentiality and allow questions before proceeding

In this session we'll be discussing [Name of Participant]'s [ART/PrEP] use and ways that you may support her to take this medication every day. It is very important that you do not share the information we discuss today with anyone besides [Name of Participant] unless she gives you permission. Specifically, it is extremely important that you do not disclose [Name of Participant]'s [if applicable: HIV status] or [ART/PrEP] to anyone else unless she says it is okay.

Sometimes, this disclosure can be accidental; you may be excited about what you learn here about [ART/PrEP] and want to share this information with others. If this is the case, it is imperative that you don't share the context in which you learned this information. That is, do not disclose that you learned this information in connection with [Name of Participant]'s care or this program.

Using [ART/PrEP] or [if applicable: living with HIV] is nothing to be ashamed of, but sharing this information with people can lead to reactions that someone may not want or may not be ready to deal with. For these reasons, it's extremely important that [Name of Participant] be able to choose if she wants to share this information with anyone else, and who to share it with.

Do you agree to keep this information confidential?

Do you have any questions about this before we move on?

2. EDUCATION ABOUT HIV AND ART/PREP

Review ART/PrEP information table

*Refer to participant education materials and procedures. Note to the participant (if present) that this will be a review for them. Examples from the Tonse Pamodzi 2 trial are included in the Appendices.

Afterwards, ask if the omukhulupilira has any questions before moving on.

3. THE ROLE OF THE OMUKHULUPILIRA

Define expectations of omukhulupilira

Now that you know about [ART/PrEP] and why it's so important to take it every day, let's talk about what you are being asked to do to support [Name of Participant]'s adherence to [ART/PrEP].

There is no one right way to support adherence to [ART/PrEP], and so you are being asked to be part of a collaborative process to find the best strategies for [Name of Participant] to maintain adherence centered around her individual needs and preferences. Because every person is different, these strategies will look different for each person. So, while we can't tell you exactly what the strategies will be, we can tell you what the basic expectations will be of you as an omukhulupilira. These are:

- *To help [Name of Participant] put into practice the adherence strategies she develops with the study counselor, and provide any other support appropriate to help her take [ART/PrEP] every day.*
- *Depending on [Name of Participant]'s needs, she may invite you to participate in up to 3 adherence counseling sessions to help her develop strategies to overcome problems she might be having with adherence.*

Seek agreement to serve in this role

I want to take this opportunity to see if you think you are able to play this role:

- *Do you think you will be able to meet with [Name of Participant] regularly outside of these sessions to provide this support?*
- *Do you think you will be able to meet with her in a private location?*
- *What types of places/times might you meet?*

Given what we've discussed, are you willing to serve as [Name of Participant]'s omukhulupilira?

Discuss importance of adherence support and common adherence barriers

The following includes additional education, to be conducted in a participatory manner. Try to engage the omukhulupilira through a series of questions and answers to better engage them in the topics covered.

First let's talk about why your support is so important to help [Name of Participant] adhere to [ART/PrEP]. Can you tell me about something that you have to do or have had to do on a daily basis that might seem simple but is actually difficult to keep doing every day?

[If participant has no ideas, prompt to think about common tasks. For example, for men, this may include finding work in order to provide for the family. For women, this may focus on cooking for the family.]

Would you tell me why it [is/was] difficult to do every day?

What do/did you do to make it easier, if anything? Did anyone help you?

As you can see just because a task might seem simple, like taking a pill, if we have to do it every day it might become difficult to maintain. Finding ways to overcome these difficulties, often with the help of family or friends, is essential to being able to complete the task every day.

While everyone is different, there are many common difficulties that people may face in being able to take [ART/PrEP] on a daily basis include [give examples from table below]*:*

Common barriers to PrEP adherence	Common barriers to ART adherence
• Disruption in routine<br>• Dislike of medication (too big, tastes bad)<br>• Lack privacy<br>• Scared others will think HIV+<br>• Side effects<br>• Feeling down/sad (not caring about protecting self)<br>• Feeling not at risk<br>• Memory or organization problems<br>• Alcohol/drugs	• Disruption in routine<br>• Dislike of medication (too big, tastes bad)<br>• Lack privacy<br>• Scared others will find out HIV+<br>• Side effects<br>• Feeling down/sad (not caring about protecting health)<br>• Memory or organization problems<br>• Alcohol/drugs<br>• Feel sick<br>• Feel healthy

Now let's talk about how support from a [family member/partner/friend] like you would help someone overcome these barriers. First let's take the example of forgetting – if [Name of Participant] were having trouble remembering to take her medication on time every day, what are some things you would do to help her, or advice you would give her?

Thank you, now let's take the example of feeling sad, down, or discouraged – if [Name of Participant] were feeling this way, what are some things you would do to help her?

Allow for questions

Do you have any questions about this before we move on?

4. WAYS OF PROVIDING SUPPORT FOR PrEP/ART ADHERENCE

The right way to support someone will depend on their needs and the relationship you have with them, but it may be helpful to think through some of the basic ways you can provide meaningful support. These fall into 3 basic categories:

1) *Emotional support, or providing expression of empathy, acceptance, love, trust, or care*
2) *Instrumental support, or providing tangible aid or service*
3) *Informational support, or providing advice, suggestions, or information*

Let's talk about each of these in more detail:

Discuss emotional support and empathy

Emotional support (Expressions of empathy, acceptance, love, trust, and care)

Before we talked about one example of providing support for someone who is feeling sad, depressed, or discouraged. Can you think of any other ways of providing emotional support to help [Name of participant] maintain adherence to [ART/PrEP]? [Allow person time to respond]

Thank you for sharing that, there are a lot of ways of providing emotional support to help with adherence, but some of the most common ways include:

- *Emotional support for any source of distress*
- *Providing encouragement to take medication when the person is feeling discouraged*
- *Letting them know that you care about their health and wellbeing*

- *Letting them know that you accept them regardless of their HIV status*

Empathy

An important principle to keep in mind when providing emotional support is ***empathy****, or seeking to understand rather than judge. [Name of Participant] may share person problems and emotions with you not only as part of your existing relationship, but in your new role as omukhulupilira. When she shares these sensitive topics with you, it's important that you try to receive the information with empathy, or understanding of and sympathy for the feelings and problems that she is sharing rather than disinterest or judgement.*

- *Say for example that [Name of Participant] tells you that her husband is angry with her and has been yelling at her a lot.*
  - *Here are some examples of unhelpful ways of responding:*
    - [Disinterested] *"That happens to me all the time it's part of life, you just have to deal with it."*
    - [Judgmental] *"You must have done something to make him angry, you should be more careful in the future."*
  - *Would you tell me what is wrong with both of these responses?* [Allow person time to respond]
- *Can you think of a more positive and empathetic way of responding?* [Allow person time to respond] *Thank you for sharing that example, here are a couple of others:*
  - *I'm so sorry to hear that you're going through that. I'm here to help.*
  - *This must be really difficult, thank you for sharing with me.*

Discuss instrumental support and non-punitive support

Instrumental support (Tangible aid and service)

Before we talked about one example of providing support for someone who has difficulty remembering to take their medication every day, on time. Can you think of any other ways of providing day to day, practical support to help [Name of participant] maintain adherence to [ART/PrEP]? [Allow person time to respond]

Thank you for sharing that, there are a lot of ways of providing instrumental support to help with adherence, but some of the most common ways include:

- *Help with transport or transport money to medical appointments*
- *Help picking up prescriptions if you are their guardian*
- *Providing childcare so person can attend medical appointments*
- *Bringing someone water to take their pills with*

Non-punitive support

There are some ways of encouraging [Name of Participant]'s adherence to [ART/PrEP] that may seem like good ideas but should be avoided. Specifically, using punishments like you might use for a child to encourage [Name of Participant] to take [ART/PrEP] every day is not a good approach because it may hurt her own motivation to use [ART/PrEP] by making her feel powerless. The most extreme approaches like this, such as threatening or using physical violence, will cause much more harm than good and must be avoided. Some examples of other things not to do:

- *Saying mean or hurtful things if she does not take her medication*
- *Expressing anger or yelling*
- *Threatening to or actually withholding food or other material needs if she does not take her medication*
- *Not talking to her because she did not take her medication*

- *Asking other family members for advice or to tell her that she is wrong for not taking medication*

Discuss informational support and patient-centeredness

Informational support (Advice, suggestions, and information)

The last kind of support we'll talk about is informational support, or providing advice, suggestions, or information to help a person. One major way you can help with informational support is by participating in adherence counseling sessions with [Name of Participant] to help her think of strategies to help her take [ART/PrEP] every day and on time. Can you think of any ways that you could provide advice or suggestions to her outside of these counseling sessions that could help with her adherence? [Allow person time to respond]

Thank you for sharing that, there are a lot of ways of providing informational support to help with adherence, but some of the most common ways include:

- *Medication taking reminders*
- *Daily check-in to see if took medication*
- *Reminding the person of why it is important to them to take the medication every day*
- *Help acquiring information from clinic staff regarding medication taking between appointments*

Participant-centeredness

When you provide instrumental or informational support, always keep in mind that while we may each have our own ideas about the best way to do things, it is important that we put the needs, perspective, and opinions of [Name of Participant] before our own. While your role as omukhulupilira is to help [Name of Participant] develop strategies to be able to take [ART/PrEP] every day, and help her to carry out these strategies after the counseling sessions, it's important to allow [Name of Participant] to determine with your assistance how she wants to be helped to rather than deciding on your own how you might be most helpful.

Let's talk about one example: [Name of participant] tells you she hasn't taken her medication for the past few days.

- *Here is an example of a handling the situation that puts your ideas before hers: "That's not good, I know that taking your medication every day can be hard but what you really need to do is be sure to take your pill every day when you eat dinner so you will not forget." What's wrong with this response?* [Allow person time to respond]
- *In contrast, here's a way of supporting the person that puts their needs and ideas first: "What are the reasons that you weren't able to take your medication? I'm sorry to hear that, what do you think would make this easier? What can I do to help you?"*

Allow for questions

Do you have any questions about this before we move on? [Give the person time to respond]

5. FACILITATING INITIATION OF SUPPORT

At this point I hope you understand the basic ways that you can help [Name of participant] as an omukhulupilira. Do you have any questions for me at this point?

Plan for private meetings between omukhulupilira and participant

Meeting with participant and confidentiality

Before we talked about the importance of confidentiality, or keeping sensitive information private. Would you tell me what you remember about this? [Allow person time to respond]

Yes, it's very important to not talk about the following things to other people unless [Name of Participant] gives you permission

- *HIV status*
- *Her use of [ART/PrEP]*
- *Her and your participation in this program*
- *Discussions related to these topics*

To preserve the confidentiality of this information, it's important to think through if and how you will be able to meet with [Name of Participant] privately so you can talk about [ART/PrEP] without anyone hearing your conversation who she would not want to have hear it.

[Involve participant in discussion if present]

- *Where do you think you can meet to speak comfortably in private, so that people will not overhear?*
- *When would you be able to meet in private?*

[If participant not present]

- *How do you think you will make a plan with her for these private meetings?*
- *When will you do this?*

Develop action steps for first support discussion with participant

Having the first support conversation (if participant present)

Now that you have a meeting plan, let's talk about how you can get started supporting [Name of participant]

Desired support: [Ask of participant]

- *Do you have any initial ideas of how [Name of Supporter] could be most helpful to support your adherence to [ART/PrEP]?* [Allow person time to respond, provide suggestions if needed]
- *How have you provided support to each other in the past?*
- *How could you apply that to this situation?*

Support to offer: [Ask of Supporter]

- *What do you think are the main ways, including or in addition to those already mentioned, that you feel able to support [Name of participant]?*

Planning future check-ins: [To both]

- *Now that you have this understanding, what do you think it would be most helpful to talk about next time you are able to speak to each other comfortably about [Name of participant] taking [ART/PrEP]?*

Planning the first support conversation (if participant *not* present)

Now that you have a good idea of how and when you will meet with [Name of Participant], let's talk about how to have the first conversation about supporting her use of [ART/PrEP].

Questions to ask:

- *What questions would you ask of [Name of participant] to better understand how she would want to be supported?*
- *Those are all great ideas; which two questions do you think will be the most important for you to ask [Name of participant] first?*

Support to offer:

- *Great, these questions will help you figure out how you can best help [Name of Participant]. What are some types of support that you could offer to her during this conversation?* [Allow person time to respond, provide suggestions if needed]

Planning future check-ins:

- *Once you understand how [Name of Participant] wants to be supported, how will you decide when to talk next at a private setting and time?*

Allow for questions

Do you have any concerns about having your [first/next] conversation? [Allow person time to respond, trouble-shoot issues that arise]

6. CLOSING THE SESSION

Thank for participation

Thank you very much for coming today and for your active participation in our session today.

Allow for final questions

Before we close the session, do you have any other questions or concerns? [Allow person to ask questions and provide responses, for questions that you are unable to answer, ask for their contact information and say that you will follow-up with an answer, or you can provide the response at the next visit]

Provide contact information

Don't hesitate to contact us if you have any other questions or concerns about your role as omukhulupilira [Provide contact information]. *You can also discuss any questions or concerns if you come with [Name of Participant] to her next counseling session.*

Thank you again for your participation and for agreeing to serve as an omukhulupilira. *Please discuss with [Name of Participant] if you should attend the first counseling session on* [*date and time of scheduled appointment*]. *This decision will be based on her needs and your availability.*

ORIENTATION VISIT CHECKLIST

Date (DD/MMM/YYYY) |___|___|/|___|___|___|/|___|___|___|___| Start time ___ ___: ___ ___

Participant ID |___|___|___|___|___| Staff ID |___|___|___|

Omukhulupilira gender: ☐ Male ☐ Female

Omukhulupilira relationship with participant: ____________________

Participant present? ☐ Yes ☐ No

◯ **1. Introducing the counseling visit**
- ☐ Check in and build rapport
- ☐ Confirm omukhulupilira identity (if participant not present)
- ☐ Ascertain disclosure of HIV-status (if applicable) and ART/PrEP use
- ☐ Provide session overview
- ☐ Discuss importance of confidentiality
- ☐ Allow for questions

◯ **2. ART/PrEP education**
- ☐ Review ART/PrEP information table
- ☐ Allow for questions

◯ **3. Role of the omukhulupilira**
- ☐ Define basic expectations of omukhulupilira
- ☐ Seek assent to play this role
- ☐ Discuss importance of adherence support and common adherence barriers
- ☐ Allow for questions

◯ **4. Ways of providing support for PrEP/ART adherence**
- ☐ Discuss Emotional support, empathy
- ☐ Discuss Instrumental support, non-punitive support
- ☐ Discuss Informational support, person-centered support
- ☐ Allow for questions

◯ **5. Facilitating initiation of support**
- ☐ Plan for private meeting omukhulupilira and participant
- ☐ Develop action steps for first support discussion with participant
- ☐ Allow for questions

◯ **6. Closing session**
- ☐ Thank for participation
- ☐ Allow for final questions
- ☐ Provide contact information

Brief description of the mood, content, and dynamics of the session; things to remember for next visit:

____

____

____

____

End time ___ ___: ___ ___

SESSION COMPONENTS

Participants assigned to the intervention condition will have follow-up iNSC sessions during Month 1 and 3 visits. The aim of these visits is to follow up on the goals set during the previous iNSC sessions, first individually and then—if the participant is accompanied—with the omukhulupilira. During this part of the visit, participants will be asked to engage in the following four general phases of interaction:

1. Welcome
2. iNSC
3. Joint session with omukhulupilira (if accompanying participant)
4. Close session with plan for next steps

1. WELCOME

The session begins with a general welcome and an overview of the discussion.

> *Welcome and thank you for coming to this follow up visit. I look forward to hearing about how things went these past weeks! I also want to learn more about your journey on ART/PrEP.*

The intervention counselor should note whether the participant has chosen an omukhulupilira and whether this individual has accompanied her to the visit. This will help to frame the discussion in the iNSC component. The participant should also be given the option of inviting the omukhulupilira to join the counseling session following the individualized iNSC discussion.

2. iNSC

Similar to the enrollment visit, the iNSC component of the session includes two parts. The first part is a broader discussion about general well-being. The second part focuses in on use of [ART/PrEP], including challenges and facilitators of adherence. This is a direct discussion about how to achieve and maintain medication adherence. All intervention counselors will train in the conduct of iNSC, using the iNSC Workbook (see Appendix). The iNSC discussion structure is shown in the table below.

Step	Description
Introduce	Explain what you want to discuss, why, and ask permission<br>Ask if it is okay to split the discussion into two parts -- first about general well-being and then about adherence.<br>*Steps below will be repeated for each component.*
Review	Check in on previous goals/discussions, close and move into current experiences
Explore	Discuss socio-ecological factors that challenge or could optimize a specific behavior
Tailor	Reflect on context and experiences shared to tailor remainder of the discussion
Identify	Ask what would be needed to happen for the behavior (identified above) to be easier to handle or be more manageable
Strategize	Ask how the participant might consider addressing this need
Agree	Ask the participant if she would agree to try out one or more strategies to address the identified need
Transition / close	Move to a new topic and repeat the flow OR close the discussion

3. JOINT SESSION WITH OMUKHULUPILIRA

Participants accompanied by their omukhulupilira will be given the option of a joint session following the individualized iNSC session. This is voluntary. The purpose of this joint session is to engage the omukhulupilira in identifying and addressing challenges to medication adherence.

Participants who elect to have this joint session are asked—at the end of the iNSC session—to identify the issues they would like to discuss with their omukhulupilira in the room. These should be issues they are comfortable discussing with the omukhulupilira. This will serve as a guide for the session.

The joint session with omukhulupilira will be condensed to only three steps of the iNSC framework: review, strategize, and agree (see below). The intervention counselor encourages engagement of the omukhulupilira and ensures that he/she is given the opportunity to share observations and experiences in a manner that fosters joint problem-solving.

Step	Description
Review	Review the main issues identified by the participant for discussion regarding medication adherence
Strategize	Ask the *omukhulupilira* and participant how this issue might be addressed
Agree	Ask the *omukhulupilira* and participant if they would agree to try out one or more strategies to address the identified need

For participants who **did not** previously select an omukhulupilira, the option should be presented again.

> *Last time you were here we talked about the option to train someone to help you with your medication adherence. This person is called the omukhulupilira. Do you remember what this is?* [re-explain role if needed]
>
> *I know that last time you were here it didn't make sense to pick an omukhulupilira at that time. You still don't have to select an omukhulupilira but we want to give you the opportunity again each time you come in. Would it be okay with you if we take a few minutes now to discuss this option again to consider if you want to select an omukhulupilira today?*

If participant expresses interest in choosing an omukhulupilira, refer to **Enrollment Visit Section 3**. If permission not granted, then continue to closing the session.

4. CLOSE THE SESSION WITH PLAN FOR NEXT STEPS

Provide a summary of what was discussed, thank the participant and remind her of the next visit.

> *You noticed that [reiterate a key strategy from iNSC discussion] would really make it feel easier to work [ART/PrEP] into your life and that [reiterate a key strategy from iNSC discussion] is something that will help with that. You'll give that a try between now and the next time we meet.*
>
> *Thank you for talking with me. I look forward to talking again when you come in next time.*

SESSION COMPONENTS

The aim of this final visit is to identify new barriers to medication adherence and how to address them. It is also to summarize those strategies that have been used and provide the participant with some insights into continued engagement in this problem-solving approach.

1. Welcome
2. iNSC
3. Joint session with omukhulupilira (if accompanying participant)
4. Close session with plan for next steps

1. WELCOME

The session begins with a general welcome and an overview of the discussion.

> *Welcome and thank you for coming to this follow up visit. For this conversation together, I am hoping to spend some time getting to know how things have been going since the last visit. I also want to learn more about your journey on ART/PrEP.*

The intervention counselor should note whether the participant has chosen an omukhulupilira and whether this individual has accompanied her to the visit. This will help to frame the discussion in the iNSC component. The participant should also be given the option of inviting the omukhulupilira to join the counseling session following the individualized iNSC discussion.

2. iNSC

Similar to the enrollment visit, the iNSC component of the session includes two parts. The first part is a broader discussion about general well-being. The second part focuses in on use of [ART/PrEP], including challenges and facilitators of adherence. This is a direct discussion about how to achieve and maintain medication adherence. All intervention counselors will train in the conduct of iNSC, using the iNSC Workbook (see Appendix). The iNSC discussion structure is shown in the table below.

Step	Description
Introduce	Explain what you want to discuss, why, and ask permission<br>Ask if it is okay to split the discussion into two parts -- first about general well-being and then about adherence.<br>*Steps below will be repeated for each component.*
Review	Check in on previous goals/discussions, close and move into current experiences.
Explore	Discuss socio-ecological factors that challenge or could optimize a specific behavior
Tailor	Reflect on context and experiences shared to tailor remainder of the discussion
Identify	Ask what would be needed to happen for the behavior (identified above) to be easier to handle or be more manageable
Strategize	Ask how the participant might consider addressing this need
Agree	Ask the participant if she would agree to try out one or more strategies to address the identified need
Transition / close	Move to a new topic and repeat the flow OR close the discussion

3. JOINT SESSION WITH OMUKHULUPILIRA

Participants accompanied by their omukhulupilira will be given the option of a joint session following the individualized iNSC session. This is voluntary. The purpose of this joint session is to engage the omukhulupilira in identifying and addressing challenges to medication adherence.

Participants who elect to have this joint session are asked—at the end of the iNSC session—to identify the issues they would like to discuss with their omukhulupilira in the room. These should be issues they are comfortable discussing with the omukhulupilira. This will serve as a guide for the session.

The joint session with omukhulupilira will be condensed to only three steps of the iNSC framework: review, strategize, and agree (see below). The intervention counselor encourages engagement of the omukhulupilira and ensures that he/she is given the opportunity to share observations and experiences in a manner that fosters joint problem-solving.

Step	Description
Review	Review the main issues identified by the participant for discussion regarding medication adherence
Strategize	Ask the *omukhulupilira* and participant how this issue might be addressed
Agree	Ask the *omukhulupilira* and participant if they would agree to try out one or more strategies to address the identified need

4. CLOSE SESSION WITH PLAN FOR NEXT STEPS

Provide a summary of what was discussed. Remind the participant that this is the last scheduled intervention session and review the main issues identified and addressed through the counseling sessions. Discuss ways in which these could be applied for continued medication adherence following the study.

> *As you know, this is the final scheduled visit for the intervention part of this study. Through this process, you identified strategies that could improve your adherence. These include [reiterate key strategies from the iNSC discussions] that would make it feel easier to work [ART/PrEP] into your daily routine. I would encourage you to continue trying these ways to ensure that you take medicines on a daily basis.*

Discuss future steps for continuing ART/PrEP use, including transition to the standard of care. Details of this discussion and guidance will be site-specific but should include:

- For women living with HIV on ART:
  - Encourage women to discuss their perceived challenges about transitioning to standard of care and how those issues might be addressed.
  - Provide information and resources about continuing support for ART adherence as appropriate.
- For HIV-negative women on PrEP:
  - Discuss desire to use PrEP after study close, either in the immediate or future. Provide information about local PrEP resources.
  - Encourage women to discuss their perceived challenges about transitioning to standard of care PrEP services and how those issues might be addressed.
  - Provide information and resources about continuing support for PrEP adherence as appropriate.

Close by thanking them for their participation.

> *I hope your participation has been helpful. Thank you again for talking with me.*
